# OD44 Consolidated Health Economic Evaluation Reporting Standards For Interventions That Use Artificial Intelligence (CHEERS-AI)

**DOI:** 10.1017/S0266462324001715

**Published:** 2025-01-07

**Authors:** Tuba Saygin Avsar, Jamie Elvidge, Claire Hawksworth, Saskia Knies, Antal Zemplenyi, Zsuzsanna Petykó, Pekka Siirtola, Gunjan Chandra, Divya Srivastava, Alastair Denniston, Anastasia Chalkidou, Julien Delaye, Petros Nousios, Manuel Gomes, Junfeng Wang, Stavros Petrou, Dalia Dawoud

## Abstract

**Introduction:**

Progress and innovation in artificial intelligence (AI)-based healthcare interventions continue to develop rapidly. However, there are limitations in the published health economic evaluations (HEEs) of AI interventions, including limited reporting on characteristics and development of algorithms. We developed an extension to the existing Consolidated Health Economic Evaluation Reporting Standards (CHEERS) to improve consistency, transparency, and reliability of the reporting of HEEs of AI interventions.

**Methods:**

The Delphi method was used, following a prespecified study protocol. A steering group with expert oversight was formed to guide the development process. A long list of potential items was defined based on two recent systematic reviews of HEEs of AI-based interventions. The steering group identified and invited 119 experts to the three-stage survey. Participants were asked to score each item on a nine-point Likert scale, and they were also able to provide free-text comments. The final checklist was piloted on a random sample of nine HEEs of AI-based interventions.

**Results:**

Three stages of the Delphi survey were completed by 58, 42, and 31 multidisciplinary respondents, respectively, including HTA specialists, health economists, AI experts, and patient representatives. The CHEERS-AI extension includes 18 AI-specific reporting items. Ten are entirely new items, including considerations about user autonomy, validation of the AI component, and AI-specific uncertainty. In addition, elaborations on eight existing CHEERS items were added to emphasize important AI-specific nuances. Some participants highlighted that CHEERS-AI can provide key benefits; for example, it could clarify the misconception that the predictive algorithms supporting AI-driven healthcare interventions are available for use without cost.

**Conclusions:**

CHEERS-AI can aid in improved reporting quality for researchers, editors, and reviewers conducting or assessing HEEs of AI interventions.

